# Prevalence and risk factors of myopia in Han and Yugur older adults in Gansu, China: a cross-sectional study

**DOI:** 10.1038/s41598-020-65078-x

**Published:** 2020-05-19

**Authors:** Xuqian Wang, Huijing He, Xuejiao Wang, Guangliang Shan, Zhiyan Tao, Li Pan, Jun Li, Xiaolan Ren, Hongjun Zhao, Zhouxian Pan, Meng Wang, Yong Zhong, Jin Ma

**Affiliations:** 1 Department of Ophthalmology, Peking Union Medical College Hospital, Chinese Academy of Medical Sciences & Peking Union Medical College, Beijing, China. 1# Dongdan, Shuaifuyuan, Beijing, 100730 China; 2 Department of Epidemiology and Statistics, Institute of Basic Medical Sciences, Chinese Academy of Medical Sciences & Peking Union Medical College, Beijing, China. 1# Dongdan, Shuaifuyuan, Beijing, 100730 China; 3 Department of Ophthalmology, First People’s Hospital of Gansu, Gansu province, China. 1# Wujiayuan, Lanzhou, Gansu Province 730000 China; 4 Gansu Center for Disease Prevention and Control, Gansu Province, China. 230# Donggangxilu, Lanzhou, Gansu Province 730000 China

**Keywords:** Refractive errors, Epidemiology

## Abstract

Few studies have investigated the prevalence of myopia in Northwest China. This cross-sectional study aimed to investigate the prevalence and associated factors of myopia and high myopia in adults aged 40–80 years in the Han and Yugur populations living in Gansu Province, Northwest China. A total of 3,845 participants were included. The overall age- and sex-adjusted prevalence of myopia (spherical equivalent (SE) < −0.5 D), high myopia (SE < −6.0 D) and hyperopia (SE > + 0.5 D) were 16.4%, 0.7% and 26.2% in Yugur participants, respectively, and 34.3%, 5.0% and 19.2% in Han participants, respectively. The prevalence of myopia and high myopia in Han participants was significantly higher than that in Yugur participants (both *P* < 0.001). Yugur population, birth in rural areas, smoking history and outdoor work were found to be negatively associated with myopia. Higher education level and a family history of myopia were found to be positively associated with myopia in the study population. High myopia was negatively associated with Yugur population, aging, birth in rural areas and was positively associated with a family history of myopia. This study provided valuable information regarding the environmental risk factors of myopia and revealed an ethnic disparity in the prevalence of myopia in Gansu Province, Northwest China.

## Introduction

Refractive error, especially myopia, is a common ocular abnormality. Complications associated with high myopia have become an important cause of low vision in adolescents and adults^1,2^. The prevalence of myopia differs by region. Many studies have shown that the prevalence of myopia is high in East Asia^3–6^. In China, the prevalence of myopia among school-age children in urban areas is 12.7–35.8%^7,8^ and 70–87.7%^8–10^ in people aged over 17. Although some studies explored that the prevalence of myopia among Chinese immigrants was the highest than other ethnicities of their place of residence^11–13^, the genetic susceptibility on the high prevalence of myopia in Chinese population is not sufficient as their cultural attitudes to education sustained impact the learning pressure. Previous studies have also indicated that myopia was more related to environmental factors (early learning^14^, near work^15^, and lack of outdoor activities^16,17^) than genetic factors.

China is a multiethnic country composed of the Han ethnicity and 55 other populations, which results in differences in health and clinical profiles. The disparities in the prevalence of myopia in different populations in China have been studied in teenagers^18,19^. The prevalence of myopia among subjects aged 4–19 years of the Han population (27%) was significantly higher than those of the Hui (18%) and Uyghur (13%) living in Turpan, China^19^. One study among Han and Yi adults in Yunnan Province showed that the Yi population had a lower prevalence of myopia (8.1% vs. 10.3%; *P* = 0.02)^20^. Disparities in myopia susceptibility may be attributed to multiple factors, such as geographic locations, lifestyle factors and genetic backgrounds. Among the previous epidemiological studies, the population has been from well-developed areas in East China (except for the Yunnan minority eye study^20^), with no exploration on the Yugur population. Our study is the first to investigate the prevalence of myopia and high myopia in the Yugur population, who live in Gansu Province.

Gansu Province is in Northwest China, with an area of 424,900 square kilometers and a population of 25,575,000 (the data were based on the sixth population census of China in 2010)^21^. The Yugur population is a unique minority in Gansu Province. This was the main consideration when we chose the representative minorities in Gansu Province. Yugur population has been considered to be the descendants of ancient Huihu people and Mongols^22,23^. In the 13th century, Huihe individuals living around the Hexi Corridor and some Mongolians gradually formed one new group, the Yugur. Furthermore, the Tibetan culture and the Yugur group’s surrounding ethnic groups, such as the Tu population, also contributed to the current formation of the Yugur group^24^. The Yugurs is a population mainly engaged in animal husbandry, with a total population of 1,438,721 in 2010^21^. The Yugur and Han populations have distinct living environments and habits. The diversity of the ethnic populations may contribute to the disparity in disease prevalence as well as associated factors. In our study, data on adult myopia and high myopia was derived from the China National Health Survey (CNHS), which has been described elsewhere^25^. This study aimed to address the gap in current knowledge on the prevalence of myopia among Yugur and Han adults, and provide further understanding of risk factors of myopia among population with ethnic diversity.

## Results

### Characteristics of the Han and Yugur populations

Of the entire study sample, 3,845 participants aged 40–80 years completed face-to-face questionnaire interview, physical examination and refractive error assessment. There were 2,788 Han participants (1190 men; 42.7%) and 1,057 Yugur participants (507 men; 48.0%). Compared with the Han participants, the Yugur participants spent more time in rural areas (*P* < 0.001), had lower education levels (primary school or below: men 52.1% vs. 24.5%, *P* < 0.001; women: 72.2% vs. 38.8%, *P* < 0.001), were more engaged in outdoor work (*P* < 0.001) and had higher activity levels (*P* < 0.001). More participants in the Yugur group had ever been smokers (*P* < 0.001) and had ever been drinkers (men *P* = 0.008; women *P* < 0.001) than those in the Han group. There were also differences between the Han and Yugur participants regarding the prevalence of hypertension (men: 35.3% vs. 44.4% *P* < 0.001, respectively; women: 28.5% vs. 34.9%, *P* = 0.005, respectively) and diabetes in the male group (11.9% vs. 7.3% *P* = 0.004, respectively) (Table 1).

**Table 1:** Characteristics of Han and Yugur participants in Gansu Province, China, 2016.

	Male		*P*	Female		*P*
	Han n = 1190	Yugur n = 507		Han n = 1598	Yugur =550	
Age (year)	55.1 ± 8.7	53.0 ± 8.4	<0.001	53.9 ± 8.0	52.3 ± 7.9	<0.001
Age group			<0.001			0.014
40–49	400 (33.6)	239 (47.1)		579 (36.4)	241 (43.8)	
50–59	430 (36.1)	162 (32.0)		630 (39.7)	198 (36.0)	
60–69	292 (24.5)	98 (19.3)		333 (21.0)	97 (17.6)	
70–80	68 (5.7))	17 (3.4)		56 (3.5)	14 (2.5)	
Height (cm)	168.7 ± 5.9	169.40 ± 5.7	0.021	157.5 ± 5.1	158.4 ± 5.8	0.001
Weight (kg)	69.7 ± 10.2	71.2 ± 11.6	0.008	58.8 ± 8.0	64.3 ± 12.0	<0.001
BMI (kg/m^2^)	24.5 ± 3.1	24.7 ± 3.5	0.086	23.7 ± 2.9	25.6 ± 4.2	<0.001
Birthplace			<0.001			<0.001
Urban	278 (23.4)	25 (4.9)		553 (34.6)	44 (8.0)	
Rural	912 (76.6)	482 (95.1)		1045 (65.4)	506 (92.0)	
Occupation			<0.001			<0.001
Outdoor	451 (37.9)	342 (67.5)		529 (33.1)	346 (62.9)	
Indoor	739 (62.1)	165 (32.5)		1069 (66.9)	204 (37.1)	
Time spent in rural areas (year)	32.0 ± 23.9	43.5 ± 18.1	<0.001	26.5 ± 23.8	42.6 ± 18.4	<0.001
Education			<0.001			<0.001
Primary school or below	292 (24.5)	264 (52.1)		620 (38.8)	397 (72.2)	
High school	572 (48.1)	173 (34.1)		694 (43.4)	111 (20.2)	
Undergraduate/graduate	326 (27.4)	70 (13.8)		284 (17.8)	42 (7,6)	
Hypertension	420 (35.3)	225 (44.4)	<0.001	455 (28.5)	192 (34.9)	0.005
Diabetes	142 (11.9)	37 (7.3)	0.004	89 (5.6)	21 (3.8)	0.108
Smoking status			<0.001			<0.001
Never-smoke	301 (25.8)	88 (17.4)		1588 (99.9)	519 (94.4)	
Ever-smoke	889 (74.2)	419 (82.6)		10 (0.1)	31 (5.6)	
Alcohol consumption			0.008			<0.001
Never-drink	138 (11.6)	37 (7.3)		1071 (67.0)	279 (50.7)	
Ever-drink	1051 (88.4)	470 (92.7)		527 (33.0)	271 (49.3)	
Physical activity level			<0.001			<0.001
Light	107 (9.0)	28 (5.5)		207 (13.0.)	57 (10.4)	
Moderate	882 (74.1)	340 (67.1)		1188 (74.3)	360 (65.5)	
Heavy	201 (16.9)	139 (27.4)		203 (12.7)	133 (24.2)	

Note: BMI: Body mass index was defined as weight (kg)/height (m)^2^. 1) 125 participants had missing values on refractive error data; 2) 51 participants had missing values on birth place information; 3) 65 participants had missing values on education or work information. The Chi-square test was used to evaluate the demographic and life-style factors differences between Han and Yugur participants.

### Prevalence of refractive error in the Han and Yugur participants

**Figure 1:**
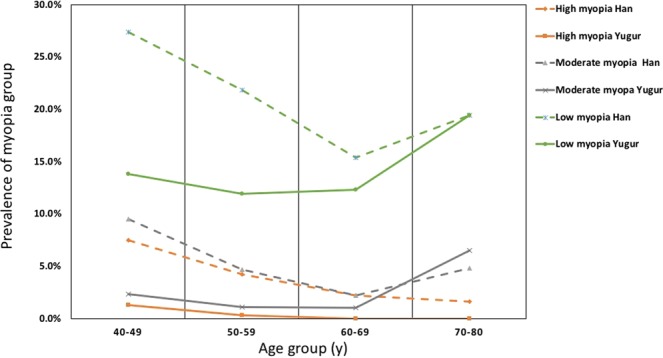
Prevalence of myopia and high myopia in different age groups in Han and Yugur populations. Adjusted-prevalence (vertical axis) of different age groups (horizontal axis) are plotted against different myopia groups in the Yugar and Han populations.

The crude sex- and age-specific prevalence of low myopia, moderate myopia, high myopia, emmetropia and hyperopia in Han and Yugur groups were presented in Table 2. The overall sex- and age-adjusted prevalence of myopia (SE < −0.5 D) and high myopia (SE < −6.0 D) were 16.4% and 0.7%, respectively, in Yugur population; and 34.3% and 5.0%, respectively, in Han population. In both populations, the prevalence of myopia decreased in 50–59 age group (*P* < 0.001) and increased in 70–80 age group (*P* = 0.041). The prevalence of high myopia decreased with age in 50–59 (*P* = 0.039) and 60–69 (*P* = 0.049) age groups (Fig. 1). Overall, compared with Han adults, Yugur adults had a lower prevalence of myopia (men: 15.8% vs. 32.1%, *P* < 0.001; women: 14.9% vs. 33.4%, *P* < 0.001) and high myopia (men: 0.2% vs. 3.7%, *P* < 0.001; women: 1.1% vs. 5.6%, *P* < 0.001). The prevalence of emmetropia was higher in the Yugur participants (men 57.0% vs. 46.8%, *P* < 0.001; women 62.0% vs. 47.9%, *P* < 0.001). Moreover, there was a small but significant difference between the prevalence of hyperopia in the Yugur and Han ethnicities in both the male and female groups (men: 26.8% vs. 21.1%, *P* = 0.012; women: 23.1% vs. 18.7%, *P* = 0.026) (Table 3).

**Table 2:** Adjusted-prevalence of myopia and other refractive errors among adults aged 40–80 in Gansu, China, 2016.

	N	Prevalence							
		Crude	P (95% CI)	Adjusted	P (95% CI)	Age group			
						40–49	50–59	60–69	70–80
Myopia									
Overall	1080	28.1%	(26.7–29.5)	28.8%	(27.4–30.3)	35.6%	26.3%	18.3%	25.8%
Han ethnicity	916	32.9%	(31.1–34.6)	34.3%	(32.5–36.1)	44.3%	30.8%	19.8%	25.8%
Yugur ethnicity	164	15.5%	(13.3–17.7)	16.4%	(14.2–18.7)	17.4%	13.3%	13.3%	25.8%
Men	464	27.3%	(25.2–29.5)	28.3%	(26.2–30.4)	33.8%	26.4%	18.5%	27.1%
Women	616	28.7%	(26.8–30.6)	29.4%	(27.4–31.3)	37.0%	26.3%	18.1%	24.3%
Low myopia									
Overall	757	19.7%	(18.4–20.9)	20.1%	(18.8–21.4)	23.0%	19.3%	14.6%	19.4%
Han ethnicity	619	22.2%	(20.7–23.7)	22.9%	(21.3–24.4)	27.4%	21.8%	15.4%	19.4%
Yugur ethnicity	138	13.1%	(11.0–15.1)	13.6%	(11.5–15.7)	13.8%	11.9%	12.3%	19.4%
Men	372	21.9%	(20.0–23.9)	20.4%	(18.5–22.4)	22.5%	20.6%	15.6%	20.0%
Women	415	19.3%	(17.7–21.0)	19.7%	(18.1–21.4)	23.3%	18.4%	13.7%	18.6%
Moderate myopia									
Overall	182	4.7%	(4.1–5.4)	5.1%	(4.4–5.8)	7.2%	3.8%	2.0%	5.2%
Han ethnicity	163	5.8%	(5.0–6.7)	6.4%	(5.5–7.3)	9.5%	4.7%	2.2%	4.8%
Yugur ethnicity	19	1.8%	(1.0–2.6)	2.2%	(1.3–3.1)	2.3%	1.1%	1.0%	6.5%
Men	77	4.5%	(3.5–5.5)	5.0%	(3.9–6.0)	7.1%	3.5%	1.5%	5.9%
Women	105	4.9%	(4.0–5.8)	5.2%	(4.2–6.1)	7.2%	4.0%	2.3%	4.3%
High myopia									
Overall	141	3.7%	(3.1–4.3)	3.6%	(3.0–4.2)	5.4%	3.2%	1.7%	1.3%
Han ethnicity	134	4.8%	(4.0–5.6)	5.0%	(4.2–5.9)	7.5%	4.2%	2.2%	1.6%
Yugur ethnicity	7	0.7%	(0.2–1.2)	0.7%	(0.2–1.2)	1.3%	0.3%	0.0%	0.0%
Men	45	2.7%	(1.9–3.4)	2.8%	(2.0–3.6)	4.1%	2.2%	1.3%	1.2%
Women	96	4.5%	(3.6–5.3)	4.5%	(3.6–5.4)	6.5%	4.0%	2.1%	1.4%
Emmetropia									
Overall	1953	50.8%	(49.2–52.4)	49.9%	(48.4–51.5)	56.2%	53.5%	40.5%	30.3%
Han ethnicity	1323	47.5%	(45.6–49.3)	46.5%	(44.7–48.4)	50.3%	51.3%	39.8%	30.6%
Yugur ethnicity	630	59.6%	(56.6–62.6)	57.3%	(54.3–60.3)	68.6%	59.7%	42.6%	29.0%
Men	846	49.9%	(47.5–52.2)	49.4%	(47.0–51.7)	57.5%	53.2%	37.9%	24.7%
Women	1107	51.5%	(49.4–53.7)	50.5%	(48.4–52.6)	55.2%	53.6%	42.8%	37.1%
Hyperopia									
Overall	812	21.1%	(19.8–22.4)	21.2%	(19.9–22.5)	8.2%	20.2%	41.2%	43.9%
Han ethnicity	549	19.7%	(18.2–21.2)	19.2%	(17.7–20.6)	5.4%	17.9%	40.3%	43.5%
Yugur ethnicity	263	24.9%	(22.3–27.5)	26.2%	(23.6–28.9)	14.0%	26.9%	44.1%	45.2%
Men	387	22.8%	(20.8–24.8)	22.3%	(20.3–24.3)	8.7%	20.4%	43.6%	48.2%
Women	425	19.8%	(18.1–21.5)	20.1%	(18.4–21.8)	7.8%	20.0%	39.1%	38.6%

Note: CI, confidence interval. Prevalence of All subjects were adjusted by age and sex. Prevalence of the Han ethnicity, Yugur ethnicity, Men and Women were adjusted by age.

**Table 3:** Crude prevalence in the Han and Yugur adults aged 40–80 in Gansu Province, China, 2016.

	Han		Yugur		*P*
	N	%*	N	%*	
Myopia					
Men	382	32.1	80	15.8	<0.001
Women	534	33.4	82	14.9	<0.001
Low myopia					
Men	270	22.7	72	14.2	<0.001
Women	349	21.8	66	12.0	<0.001
Moderate myopia					
Men	68	5.7	9	1.8	<0.001
Women	95	5.9	10	1.8	<0.001
High myopia					
Men	44	3.7	1	0.2	<0.001
Women	90	5.6	6	1.1	<0.001
Emmetropia					
Men	557	46.8	289	57.00	<0.001
Women	766	47.9	341	62.00	<0.001
Hyperopia					
Men	251	21.1	136	26.8	0.012
Women	298	18.7	127	23.1	0.026

Note: The difference in crude refractive error prevalence between the Han and Yugur ethnicities was analyzed using a general linear regression model.

### Risk factors for myopia and high myopia

The influencing factors for myopia yielded by the logistic regression model are presented in Table 4. We found that compared with the youngest age group (40–49), the age group of 50–59 was less likely to have myopia (OR, 0.71; 95% CI: 0.60–0.86, *P* < 0.001), and the age group of 70 or older was positively associated with myopia (OR, 1.64; 95% CI: 1.02–2.63, *P* = 0.041). Yugur ethnicity (OR, 0.56; 95% CI: 0.45–0.69, P < 0.001), birth in rural places (OR, 0.69; 95% CI: 0.56–0.85, *P* < 0.001), work outdoors (OR, 0.64; 95% CI: 0.51–0.80, *P* < 0.001), and smoking history (OR, 0.69; 95% CI: 0.52–0.90, *P* = 0.007) were negatively associated with myopia. Compared with the primary school or less education group, people with higher educational level had an increased risk of myopia, with an OR of 1.48 (95% CI: 1.18–1.85, *P* = 0.001) for the middle/high school group and an OR of 3.62 (95% CI: 2.73–4.82, *P* < 0.001) for the undergraduate/graduate group. There was a positive association between myopia and a family history of myopia in only one parent (OR, 2.83; 95% CI: 1.22–6.51, *P* = 0.015). However, for high myopia, the associated factors included only ethnicity (OR, 0.33; 95% CI: 0.15–0.73, *P* = 0.006), aging (OR, 0.65, 95% CI: 0.43–1.00, *P* = 0.049 in the 50–59 age group; OR, 0.49, 95% CI: 0.25–0.97, *P* = 0.039 in the 60–69 age group), birth in rural areas (OR, 0.61; 95% CI: 0.40–0.94, *P* = 0.025) and a family history of myopia (OR, 4.32, 95% CI: 1.74–10.7, *P* = 0.002 in one myopic parent group; OR, 22.30, 95% CI: 2.41–206.1, *P* = 0.006 in both myopic parents group). Neither sex, activity level, medical history of hypertension nor diabetes had a statistically significant association with myopia or high myopia in the present study.

**Table 4:** Multivariable regression of myopia and high myopia in Han and Yugur adults aged 40–80 in Gansu, China, 2016.

	Myopia				High myopia			
	Odds Ratio	95% CI lower	95% CI upper	*P*	Odds Ratio	95% CI lower	95% CI upper	*P*
Race								
Han	1.00	NA	NA	NA	1.00	NA	NA	NA
Yugur	0.56	0.45	0.69	<0.001	0.33	0.15	0.73	0.006
Age range								
40–49	1.00	NA	NA	NA	1.00	NA	NA	NA
50–59	0.71	0.60	0.86	<0.001	0.65	0.43	1.00	0.049
60–69	0.84	0.65	1.09	0.185	0.49	0.25	0.97	0.039
70–80	1.64	1.02	2.63	0.041	0.27	0.06	1.20	0.085
Sex								
Male	1.00	NA	NA	NA	1.00	NA	NA	NA
Female	0.83	0.63	1.08	0.168	1.06	0.59	1.91	0.838
Birthplace								
Urban	1.00	NA	NA	NA	1.00	NA	NA	NA
Rural	0.69	0.56	0.85	<0.001	0.61	0.40	0.94	0.025
Education								
Primary school or below	1.00	NA	NA	NA	1.00	NA	NA	NA
Middle/high school	1.48	1.18	1.85	0.001	0.59	0.31	1.15	0.125
Undergraduate/graduate	3.62	2.73	4.82	<0.001	0.62	0.30	1.30	0.205
Occupation								
Indoor	1.00	NA	NA	NA	1.00	NA	NA	NA
Outdoor	0.64	0.51	0.80	<0.001	0.57	0.28	1.15	0.117
Activity level	0.88	0.74	1.04	0.137	0.95	0.64	1.41	0.800
Smoking status								
Never-smoke	1.00	NA	NA	NA	1.00	NA	NA	NA
Ever-smoke	0.69	0.52	0.90	0.007	0.61	0.32	1.16	0.132
Hypertension								
Without	1,00	NA	NA	NA	1.00	NA	NA	NA
With	1.17	0.97	1.40	0.092	1.01	0.66	1.56	0.954
Diabetes								
Without	1.00	NA	NA	NA	1.00	NA	NA	NA
With	1.01	0.74	1.37	0.948	1.71	0.91	3.23	0.098
Myopia family history								
0	1.00	NA	NA	NA	1.00	NA	NA	NA
1	2.83	1.22	6.51	0.015	4.32	1.74	10.7	0.002
2	4.67	0.52	42.3	0.170	22.3	2.41	206.1	0.006

Note: CI, confidence interval. High Myopia was defined as SE < −6.0D. Myopia was defined as SE < −0.5D. Myopia family history: 0: no parent was myopic; 1: only one parent was myopic; 2: both parents were myopic. Activity level was defaulted to be continuous variables for statistics.

## Discussion

Our study explored the prevalence of myopia in Han and Yugur adults aged 40–80 years in Gansu Province, Northwest China. In this study, the age- and sex-adjusted prevalence of low and moderate myopia decreased with age but mildly increased in the 70–80-year age group (Fig. 1). The U-shaped curve of myopia prevalence was consistent with the findings of previous studies in China^26,27^. A birth cohort study in Europe showed that the prevalence of myopia was low for cohorts born before 1940, but there was a considerable increase in the prevalence of myopia in a more recent cohort when measured at the same age^28^. Data in this birth cohort suggested that increases in the prevalence of myopia may be due to extended education to meet the needs of modern industry^29^. Recent studies have indicated that the rapidly growing incidence of myopia in youths was mainly attributed to environmental factors, among which education was believed to be strongly associated with the increased incidence of myopia; in other words, education is a key causal factor of myopia^29^. A study conducted among German adults revealed that higher levels of school and postschool professional education are associated with more myopic refraction^30^. In our study, most elderly people who were born in prerevolutionary China received unsystematic education in a tumultuous period of Chinese history. The youngest subjects were educated in a society characterized by rapid economic development and the expansion of the education system. Therefore, the younger participants had higher educational levels than the elderly participants had and completed many more years of school. Therefore, it is reasonable to consider that younger individuals may have high levels of reading with a large amount of near work activity. In this scenario, the difference in the exposure level of environmental risk factors among generations may explain the pattern of decreasing myopia prevalence with increasing age. The increased prevalence of myopia among subjects aged 70–80 years might be related to the increased density of the crystalline lens in this group^31^. The prevalence of hyperopia increased with age in both ethnic groups. The age-related change in prevalence was also concluded in previous studies^32–35^.

As Han is the predominant ethnicity in China, surveys focusing on the prevalence of myopia in the Han population have been carried out worldwide. To make the comparison more specific, we compared the prevalence of myopia (30.8%) and high myopia (5.0%) in the 50–59 age group in the Han population with the prevalence estimates from other studies in Asia or worldwide (Table 5). Previous studies conducted in Han Chinese adults have reported that the prevalence of myopia and high myopia were higher among populations in well-developed countries (35.9–41.1%)^11,12,33,36^ but lower in less developed countries or areas where participants were recruited only from the outer suburbs and had access to lower education (10.3%)^20^. This socioeconomic disparity in the prevalence of myopia suggests that the population in more developed countries or areas may experience more intensive education and limited outdoor time, which could promote the development of myopia^37,38^.

**Table 5:** Comparison of reported prevalence of myopia and high myopia in population-based studies among older adults.

	Myopia (%+95%CI)	Myopia (SE)	High myopia (%+95%CI)	High myopia (SE)	Publish year	Population	Age	Sample size
Liwan study^25^	31.4 (25.0–37.8)	<−0.5D	6.3 (2.9–9.6)	<−5.0D	2009	Chinese	50–59	1269
The Chinese American eye study^28^	36.1 (34.1–38.2)	<−0.5D	7.4 (6.6–8.3)	<−5.0D	2017	Chinese	50–59	4144
The Singapore epidemiology of eye disease study^11^	35.9 (34.4–37.5)	<−0.5D	5.1 (4.4–5.9)	<−5.0D	2013	Chinese	50–59	3013
The Shanghai Eye Study^29^	24.0 (22.3–25.7)	<−0.5D	5.11 (4.22–5.99)	<−6.0D	2017	Chinese	50–59	5099
The Andhra Pradesh Eye disease study^36^	13.5 (11.9–15.1)	<−0.5D	1.0 (0.5–1.4)	<−5.0D	2009	Indian	50–59	3642
IDNEYE study of South Indian^37^	16.5 (13.9–19.4)	≤−0.75D	2.0*	≤−6.0D	2018	Indian	40–59	3267
Gutenberg Health Study^38^	29.3 (27.8–30.8)	<−0.5D	3.5*	−6.0~ −9.0 D	2014	Germans	55–64	13959
The Tajimi study (urban Japan) ^†^^32^	49.6 (44.8–54.4)	<−0.5D	8.7 (6.0–11.4)	<−5.0D	2008	Japanese	50–59	3021
The Kumejima study (rural Japan) ^†^^35^	29.4 (25.0–34.2)	<−0.5D	0.8 (0.3–2.3)	<−5.0D	2018	Japanese	50–59	2384
Korea national health and nutrition examination survey^34^^.^	55.2 (54.5–55.9)	≤−0.5 D	4.0 (3.8–4.2)	≤−6.0D	2019	Korean	45–49	3398
Beijing eye study^26^	22.9 (21.7–24.2)	<−0.5D	2.6 (2.1–3.1)	<−6.0D	2005	Chinese	>40	4319
The Handan Eye study^31^	18.2 (17.0–19.4)	<−0.5D	2.6 (2.1–3.1)	<−5.0D	2009	Chinese	≥50	4005
The Yunnan Minority Eye Studies (Han ethnicity)^21^	10.3 (9.0–11.7)	<−0.5D	2.4 (1.7–3.0)	<−6.0D	2015	Han	≥50	2205
The Yunnan Minority Eye Studies (Yi ethnicity)	8.1 (6.8–9.4)	<−0.5D	1.6 (0.9–2.2)	<−6.0D	2015	YI	≥50	2208
Yunnan CNHS study (Han ethnicity)^30^	31.5 (27.6–35.5)	<−0.5D	3.34 (1.75–3.53)	<−6.0D	2019	Han	40–80	1085
Yunnan CNHS study (Yi ethnicity)	16.8 (12.8–20.8)	<−0.5D	1.31 (0.19–2.43)	<−6.0D	2019	Yi	40–80	541
The Hong Kong vision study^33^	41.1	<−0.5D	8.2	<−5.0D	1997	Chinese	≥40	355
The Singapore Malay Eye Survey^39^	26.2 (26.0–26.4)	<−0.5D	3.9 (3.8–4.0)	<−5.0D	2008	Malay	40–80	2974
The Multi-Ethnic Study of Atherosclerosis (Chinese)^12^	37.2	≤−1.0D	11.8	≤−5.0D	2013	Chinese	45–84	487
The Multi-Ethnic Study of Atherosclerosis (White)	31	≤−1.0D	5.4	≤−5.0D	2013	White	45–84	1667

Note: *95%CI was not displayed; † only prevalence of men or women was available, we chose men for comparison.

Recent knowledge on the etiology of myopia tends to suggest that environmental risk factors play the predominant role in the development of myopia^29^. In our study, the prevalence of myopia in the Yugur population was significantly lower than that in Han population. In the present study, compared with Han participants, the Yugur population, most of whom were born in rural areas, have a much lower educational level and a higher proportion of outdoor work. As stated previously, a longer school year and a lower outdoor activity level are strongly associated with the risk of myopia^39–41^. Therefore, environmental factors, such as educational level and outdoor activity level, could, to some extent, explain the ethnic difference in the prevalence of myopia between the Yugur and Han populations in this study. Although no genomic study has explored differences in the susceptibility to myopia between the Yugur and Han groups, there has been study indicated that the Yugur population is closely related to the Han population and to the Mongolian population and is clearly discrete from the Tibetan and the Uygur populations genetically^42^. Thus, we suppose that the difference in the myopia prevalence between the Yugur and Han populations was more related to environmental risk factors than to genetic background. Evidence from other studies also suggests that differences in environmental exposures, rather than genetic background, are the primary cause of ethnic variation in myopia. For example, Singapore provides a multiethnic laboratory in which the role of environmental factors in generating ethnic differences can be assessed^29^. The Singapore study^11^, which included Malays, Indians and Chinese individuals aged 40–80, found that the Chinese group had the highest myopia prevalence. However, it is notable that all ethnic groups in Singapore are more myopic than the same ethnic groups in other parts of the world. For example, the prevalence of myopia in children of Indian origin is much higher in Singapore than in India^43^. Likewise, the prevalence of myopia is higher in Singapore Malays than in Malays in Malaysia^44^. A study conducted in Yunnan Province (Southwest China) among the Yi and Han populations found that the Han ethnicity was no longer associated with myopia after adjustments were made for socioeconomic characteristics in the regression model^35^.

In addition, since 2010, the prevalence of myopia among Europeans and Americans over 40 years of age has increased from that in previous decades. In the Multi-Ethnic Study of Atherosclerosis (2013) in the United States, the prevalence of myopia was 31.0% in white people^12^, while it was 25.4% in 2004^45^. The Gutenberg Health Study (2014) found a myopia prevalence of 35.1% in European adults^46^, while it was 25.4–26.6% in the 2000s^45,47^. The prevalence of myopia reported in the 2010s in the United States and Europe was close to the prevalence in East Asia in the same age group. Thus, we believe that the ethnic disparity in myopia in this study mainly resulted from different levels of environmental exposure rather than genetic backgrounds.

In the present study, we also found that smoking history was negatively associated with myopia. The nicotinic cholinergic receptor is one of the main acetylcholine receptors distributed in the retina^48^. The results of studies on the impact of cigarette smoking on myopia are inconsistent. A study including 1,334 Chinese children from three schools in Singapore found no significant association between parental smoking and refractive error^49^. The CNHS study in Yunnan Province revealed that smoking history was not associated with myopia (OR, 1.27; 95% CI: 0.76–2.13)^35^. However, several studies indicated that current smoking was negatively associated with myopia (OR, 0.7; 95% CI: 0.5–0.9)^50^. We believe that the impact of smoking on myopia development has duration and dose accumulation effects. It is possible that neither the duration nor dosage of smoking by the subjects themselves were enough to affect the development of myopia in those studies. The impact of nicotine on the development of myopia requires further laboratory and prospective studies. Furthermore, the association between smoking and myopia could also be modified by socioeconomic status (SES) and education level. SES inequalities may be linked with smoking behavior: people with lower SES and lower education level are more likely to have higher smoking prevalence^51–54^. We should consider the possibility of the impact of SES and education level on smoking behavior when discussing the association between smoking and myopia. In the present study, we obtained a statistically significant result, but a causal inference cannot be obtained because of the cross-sectional design. The conclusion of the relationship between myopia and smoking requires more exploration.

High myopia is an important cause of eye problems that need to be considered in the clinic^55^. In parallel with the epidemic of myopia, an epidemic of high myopia has appeared^29,56^. The etiology of high myopia includes genetic and environmental aspects^56^. High myopia of genetic etiology tends to be early in onset and severe. Although an increasing number of genes associated with high myopia have been found^57–60^, previous studies have indicated that the epidemic of high myopia is caused by environmental factors^56^. “Acquired” high myopia is associated with the early onset of myopia because of the early imposition of severe study pressure, which gives myopia more time to progress before it stabilizes^56,61^. In line with other studies^35,46^, the present study revealed that the prevalence of high myopia in young adults is higher than that in older groups. In this case, younger patients with high myopia who receive more education may be more likely to have an environmentally induced early onset of myopia that then progresses to high myopia. Although myopia and high myopia may share some epidemiological risk factors, the association between education and high myopia was inconsistent^62,63^. Education was not found to be associated with high myopia in some older cohorts^62^. The absence of a positive association between education and high myopia in our study may indicate that older people with relatively low educational levels are more likely to develop high myopia that is genetic in origin. The positive association between a family history of myopia and high myopia could also support this idea. Birth in rural areas was also negatively associated with high myopia. The difference in birthplace may lead to variation in environmental risk factors for myopia, such as education attainment, outdoor time, and childhood nutrition.

Strengths of our study include a large multiethnic population-based sampling strategy, a detailed questionnaire and a high response rate. The limitations of the present study should also be acknowledged. First, the nature of the cross-sectional design limited the ability of the study to conclude a causal effect regarding risk factors for myopia. Second, the study population was only older adults in Gansu Province, and thus, the external validity was limited. Third, cycloplegia refraction was not performed in our study, and ocular axial length and other biometric data were not measured; thus, we were unable to study the association between these factors and other biometric measurements and myopia. Fourth, we did not collect information on current cataract history but only cataract surgery history. Because myopia caused by cataract is quite different in etiology, prevention or treatment from that caused by axial myopia, the mixed sample may influence the prevalence as well as the estimation of the association in the present study, especially in the older group.

In summary, for the first time, we described the prevalence of myopia among adults aged 40–80 in Gansu Province, China. The prevalence of myopia in the Han population was significantly higher than that in the Yugur population. Several environmental and lifestyle factors were found to be associated with myopia, while high myopia was only associated with Han ethnicity, birthplace and a family history of myopia. These findings present a rough impression of the prevalence of myopia in Gansu Province. Our study has valuable implications for myopia prevention and control in Northwest China.

## Methods

### Study population

Our study is part of the CNHS in Gansu Province, Northwest China. The CNHS is an ongoing cross-sectional study to evaluate the national health status using a multistage cluster sampling method, conducted by the Chinese Academy of Medical Sciences^25^. Survey in Gansu Province was from June to August in 2016. The criteria for participant recruitment were: 1) aged 40–80; 2) Yugur or Han population; 3) local resident for at least one year. The exclusion criteria were: 1) women who were currently pregnant; 2) soldiers in service; 3) disabled individuals (who maybe not able to complete the whole physical examination) and 4) individuals with severe mental disorders. Considering the ethnic distribution, the Han and Yugur subjects were recruited from 6 centres: the Gansu Disease Control Centre; 3 county centres for Disease Control (Sunan Yugur autonomous county; Zhangye county; Gaotai county) and 2 village-level health centres (Lianhua village, Minghua township, Sunan county; Kangle township, Sunan county). Individuals resident in the selected areas were all invited to participate in the study.

This study was approved by the institutional review board of Institute of Basic Medical Sciences, Chinese Academy of Medical Sciences. All participants provided written informed consent.

### Measurements

All the participants were invited to the study center for a face-to-face questionnaire interview by experienced interviewers and a routine physical examination. The questionnaire contained demographic, lifestyle and health-related information, such as birthplace, current address, ethnicity, education level, occupation, smoking status, alcohol consumption, physical exercise level, medical history (day of diagnosis and treatment for diabetes and hypertension, any diagnosis or surgery history of eye diseases) and myopia family history. The physical examination included anthropometric and blood pressure measurements. Body mass index (BMI) was defined as weight (kg)/height (m)^2^. Educational level was divided into three groups (primary school or below, Middle/high school, undergraduate and above). The occupational group included outdoor and indoor work. Occupational physical labour was divided into light, medium and heavy. Exercise status was divided into 5–7 days a week (at least 20 minutes per day), 3–4 days a week, 1–2 days a week, less than 3 days a month, and never exercise. According to the degree of physical labour and exercise status, the total activity level was divided into three groups: low, moderate and high^64^. The time spent in rural areas was calculated according to the place of residence and the time at which the subjects became residents of the area. Smoking or alcohol consumption was divided into never-smoker/drinker and ever-smoker/drinker. Ever smoker/drinker included past and current smoker/drinker^25^.

### Assessment of refractive error

We performed noncycloplegic autorefraction and corneal curvature radius measurements using an auto-refractor (ARK-510A, Nidek Co., Ltd., Tokyo, Japan). An examination of the anterior segment of the eye was performed with a hand-held slit lamp (KJ5S2, Suzhou Kangjie Medical Co. Ltd., Jiangsu, China). Uncorrected visual acuity and best corrected visual acuity were measured by a logarithmic E chart (Wehen Co., Ltd., Guangzhou, China). We use spherical equivalent (SE) to evaluate the refractive error data, which was defined as a sphere plus half cylinder. In our study, emmetropia, low myopia, moderate myopia and high myopia were defined as −0.5≤ SE ≤ 0.5, −3.0≤SE < −0.5D, −3.0D < SE ≤ −6.0D, and SE < −6.0D, respectively. Hyperopia was defined as SE > + 0.5D.

### Statistical analyses

After excluding those who had eye diseases, including glaucoma, pterygium, retina disease, and eye surgical histories (including cataract surgry) or eye injury histories, the refractive error portion of the CNHS in Gansu Province included 4,599 participants aged 40 to 80 (July 2016 to August 2016). Finally, a total of 3,845 participants provided examination data and questionnaire information with no missing value on key risk factors. The response rate was 83.6%. As the correlation coefficients for SE in the left and right eye were high (Spearman correlation test, r_s_ = 0.89), only data of right eye was reported. The age- and sex- specific prevalence of low myopia, moderate myopia, high myopia and hyperopia in each ethnic group was calculated. Chi-square tests were used to compare the demographic, lifestyle-related information and physical examination data between the Han and Yugur subjects. Risk factors for myopia and high myopia were identified by multivariable logistic regression models. A *P*-value<0.05 (two-tailed) was considered statistically significant. Age- and sex-standardization was performed by direct method using the 6^th^ national census (2010) data of Chinese population as the standard population. Analyses were performed using Stata version 13.1 (StataCorp, USA) and SAS version 9.4 (SAS Institute Inc, Cary, NC, USA).

### Ethics approval and consent to participate

Our study was conducted according to the tenets of the Declaration of Helsinki. Ethics approval was received from the bioethics committee of the Institute of Basic Medical Sciences, the Chinese Academy of Medical Sciences. Written informed consent was obtained from every Han or Yugur participant.

## Data Availability

The datasets used and/or analysed during the current study are available from the corresponding author on reasonable request. This study investigated the prevalence and risk factors of myopia and high myopia in adults aged 40–80 years in the Han and Yugur populations living in Gansu Province, Northwest China.
